# Does anticoagulation needed for distally located incidental pulmonary thromboembolism in patients with active cancer?

**DOI:** 10.1371/journal.pone.0222149

**Published:** 2019-09-12

**Authors:** Haseong Chang, Min Sun Kim, Su Yeon Lee, Sun Hye Shin, Hye Yun Park, Sung-A Chang, Taek Kyu Park, Duk-Kyung Kim, Eun Kyoung Kim

**Affiliations:** 1 Division of Cardiology, Department of Medicine, Heart Vascular Stroke Institute, Samsung Medical Center, Sungkyunkwan University School of Medicine, Seoul, Korea; 2 Division of Pulmonary and Critical Care Medicine, Department of Medicine, Samsung Medical Center, Sungkyunkwan University School of Medicine, Seoul, Korea; University of Insubria, ITALY

## Abstract

**Background:**

Incidental pulmonary embolism (IPE) is frequently detected in of cancer patients undergoing CT scans for staging work up or treatment response evaluation. Nevertheless, the optimal management of IPE remains unknown. Thus, we aimed to evaluate the clinical manifestations of IPE in cancer patients and to compare the clinical prognosis according to anticoagulation therapy.

**Methods:**

We retrospectively analyzed medical records of cancer patients with newly diagnosed PE between March 2010 and December 2013. Baseline demographics, comorbidities, cancer status and clinical presentation of PE were recorded. We compared all cause death, recurrent venous thromboembolism and clinically relevant bleeding events in those with PE. Survival analysis was performed to assess effect of anticoagulation on IPE.

**Results:**

Among 703 cancer patients diagnosed with PE, IPE was identified in 474 (67.3%) patients. Compared to symptomatic patients, those with IPE had more advanced malignancy, were more likely to be on current chemotherapy at the time of IPE diagnosis. These patients tend to have smaller embolic burden, as demonstrated by the lower rate of bilateral lung involvement and RV dysfunction. While symptomatic PE showed better survival with anticoagulation (median survival 6.0 vs. 17.3 months, p = 0.003), anticoagulation did not result in significant survival benefit in IPE (median survival 15.1 vs. 21.3, p = 0.225). However, in subgroup analysis, there was significant improvement in survival with anticoagulation in patients with proximal IPE (median survival 12.2 vs. 23.4 months, p = 0.023), but not in patients with distal IPE (21.2 vs. 15.1, p = 0.906).

**Conclusions:**

In cancer patients who were diagnosed with IPE, the overall survival was different according to the embolic burden and anticoagulation therapy.

## Introduction

Cancer patients have increased thrombotic risk because of the presence of malignancy itself and secondary to chemotherapy. For these reasons, the prevalence of venous thromboembolism (VTE) increases with disease status and is reported to be 4–7 times higher in cancer patients than in those without cancer [1, 2]. Over the past several years, increasing incidence of unsuspected or incidentally detected pulmonary thromboembolism (PE) has been reported in cancer patients [3]. This is not only because cancer patients frequently undergo serial computed tomography (CT) to assess treatment response and recurrent disease, but also due to improved imaging technologies. Especially, the introduction of multi-row detector CT scanners has allowed detection of small emboli in the distal pulmonary arteries. The prolonged survival introduced by the evolution of anti-cancer therapy has also posed new challenges regarding treatment of incidental PE (IPE). Notably, VTE is known to be second leading cause of death in cancer patients [4]. Therefore, establishing indication and regimen for treatment of VTE including PE is an important issue in cancer patients.

Recent guidelines for management of PE uniformly recommend that patients with active cancer should be treated for IPE regardless of its location and extent, but these recommendations are mostly based on the expert opinion rather than robust data from the randomized trials [5–7]. Due to the lack of clearly established guideline, the decision to treat IPE mainly depends on individual preference of physicians, especially in cases of distally located IPE. Furthermore, concerns regarding the bleeding risk and the potential for anticoagulants to interact with anti-cancer drugs make physicians to be more reluctant to treatment of PE. Real-world retrospective data showed that the actual anticoagulation rate of IPE in cancer patients was less than 50%, reflecting this confusing practice [8]. In this study, we aim to evaluate the clinical manifestations of IPE in cancer patients and to compare the clinical prognosis according to anticoagulation therapy.

## Materials and methods

### Study population and design

**Fig 1:**
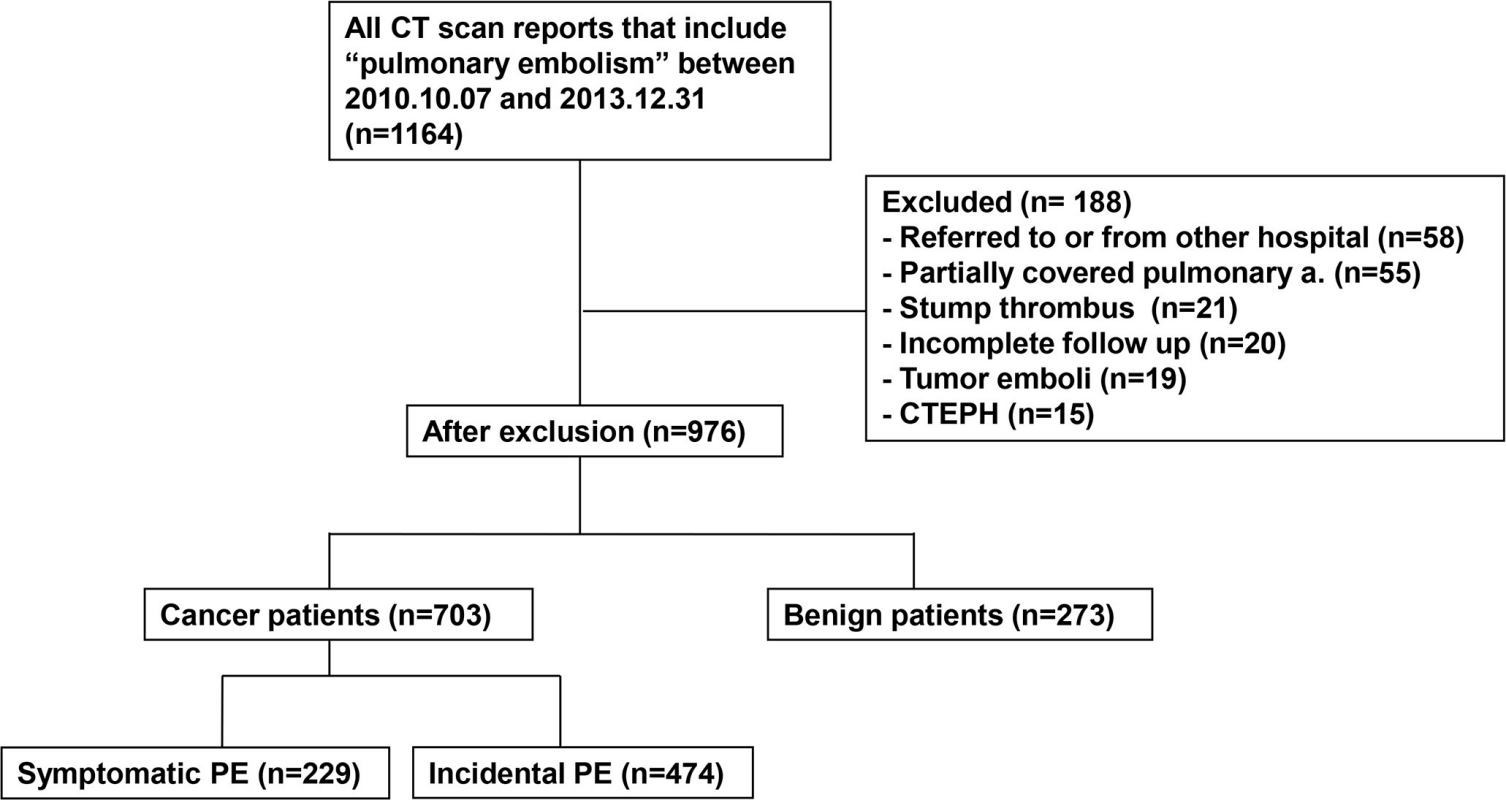
Flowchart of eligible patients. Abbreviations: CT, computed tomography; CTEPH, chronic thromboembolic pulmonary hypertension, PE, pulmonary embolism.

The study cohort consisted of consecutive patients diagnosed with PE in a tertiary referred center between October 2010 and December 2013. Among 1,164 patients diagnosed with PE, those referred from other hospital (n = 58), diagnosed with PE by abdomen CT scan that partially covered pulmonary arteries (n = 55), with filling defect in pulmonary artery stump site after lung cancer surgery (n = 21), who were lost to follow-up (n = 20), or who were found to have tumor emboli (n = 19) were excluded (Fig 1). Patients were confirmed to have cancer-related PE when they had recurrent or progressive cancer or any malignancy that required curative or palliative treatment at the time of PE detection. Both solid and hematologic malignancies were eligible. Symptomatic PE was defined as PE detected on CT scan that was ordered because of symptoms suggestive of PE. IPE is defined as a pulmonary embolism detected on CT scan performed without suspicion of PE. Clinical, laboratory, imaging findings and information regarding underlying cancer status were collected. The local Institutional Review Board of Samsung Medical Center approved this study and waived the requirement for written informed consent.

On chest CT imaging, PE was defined as the existence of contrast filling defects in one or more branches of the pulmonary artery. Location of PE and associated lung infarction were assessed. Proximal PE was defined as PE involving main or lobar level of pulmonary arteries. Distal PE was defined as PE involving segmental or subsegmental level of pulmonary arteries. Lower extremity deep vein thrombosis (DVT) was diagnosed using Doppler ultrasonography or indirect CT venography. Proximal DVT was defined as DVT occurring in the popliteal vein and above. Presence of right ventricular (RV) dysfunction was assessed using two-dimensional echocardiography.

### Clinical outcomes and definitions

We analyzed all-cause mortality and recurrent VTE events among cancer patients with PE. Clinically significant major and minor bleeding events during anticoagulation were also assessed. Major bleeding was defined according to the International Society on Thrombosis and Hemostasis criteria as fatal bleeding or clinically overt bleeding with a decrease in hemoglobin level of at least 2 g/dL or transfusion of at least 2 units of packed red blood cells occurring at a critical site (intracranial, intraspinal, intraocular, retroperitoneal, pericardial, intraarticular, and intramuscular with compartment syndrome) [9]. All non-major bleeding was considered minor bleeding.

### Statistical analyses

Baseline characteristics were summarized with continuous variables and expressed as mean ± standard deviation or median with interquartile range. Categorical data were presented as a percentage and the number of events. Continuous variables were analyzed using an independent t-test or Wilcoxon rank sum test, and categorical variables were analyzed using a chi-square test or Fisher’s exact test. Multivariate model with backward elimination was performed to identify independent factors which associated with PE prognosis. Factors included in the multivariate analysis were treatment with anticoagulation, initial vital sign, location of PE, presence of PE-related symptoms and baseline cancer status such as metastasis, progressive disease and chemotherapy.

Kaplan-Meier survival analysis was used to compare survival between groups. All tests were two-sided, and a P value < 0.05 was considered significant. All statistical analysis was performed using R Statistical Software (version 3.3.1; R Foundation for Statistical Computing, Vienna, Austria).

## Results

Among 703 patients who were confirmed to have cancer-related PE, 474 patients were classified as IPE group. Lung cancer (44.8%) was the most prevalent malignancy, followed by colon cancer (8.7%), gynecological malignancy (7.3%), and lymphoma (5.8%). Most patients had advanced stage cancer with distant metastasis (68.4%). There was no significant difference in the baseline characteristics between the patients with symptomatic and incidentally detected PE (Table 1). Duration of active malignancy did not differ between the two groups. At the time of diagnosis of PE, response to anti-cancer therapy was better in the IPE group (54.2% vs. 28.4%, p<0.001) even though the IPE group was more likely to have distant metastasis than the symptomatic PE group (71.7% vs. 61.6%, p = 0.009). The proportion of patients currently on chemotherapy was higher in the IPE group (65.8% vs. 45.9%, p<0.001).

**Table 1:** Baseline characteristics.

	Suspected PE (*n* = 229)	Incidental PE (*n* = 474)	Total (*n* = 703)	*P*-value
Male	118 (51.5%)	279 (58.9%)	397 (56.5%)	0.079
Age (years)	62.8 ± 12.6	61.9 ± 11.9	62.2 ± 12.1	0.394
Height (cm)	162.0 ± 9.2	162.4 ± 8.9	162.3 ± 9.0	0.599
Weight (kg)	61.6 ± 11.2	61.6 ± 11.5	61.6 ± 11.4	0.973
BMI (kg/m^2^)	23.4 ± 3.3	23.3 ± 3.5	23.4 ± 3.5	0.773
Diabetes	41 (17.9%)	62 (13.1%)	103 (14.7%)	0.114
Hypertension	78 (34.1%)	157 (33.1%)	235 (33.4%)	0.871
Chronic kidney disease	18 (7.9%)	21 (4.4%)	39 (5.5%)	0.092
Malignancy				
Primary site, n (%)				<0.001
Lung	90 (39.3%)	225 (47.5%)	315 (44.8%)	
Gastrointestinal	28 (12.2%)	89 (18.8%)	117 (16.6%)	
Genitourinary	28 (12.2%)	51 (10.8%)	79 (11.2%)	
Hepatobiliary & pancreatic	21 (9.2%)	47 (9.9%)	68 (9.7%)	
Hematologic	24 (10.5%)	26 (5.5%)	50 (7.1%)	
Others	38 (16.6%)	36 (7.6%)	74 (10.5%)	
Metastatic, n (%)	141 (61.6%)	340 (71.7%)	481 (68.4%)	0.009
Cancer duration (months)	15.3 ± 24.1	15.7 ± 23.5	15.6 ± 23.7	0.831
Progressive disease, n (%)				<0.001
Yes	85 (37.1%)	139 (29.3%)	224 (31.9%)	
No	65 (28.4%)	257 (54.2%)	322 (45.8%)	
Unknown	79 (34.5%)	78 (16.5%)	157 (22.3%)	
Current chemotherapy, n (%)	96 (41.9%)	312 (65.8%)	408 (58.0%)	<0.001
Radiotherapy, n (%)				0.374
Current	6 (2.6%)	7 (1.5%)	13 (1.8%)	
Past	17 (7.4%)	27 (5.7%)	44 (6.3%)	
Never	206 (90.0%)	440 (92.8%)	646 (91.9%)	

Data are presented as n (%) or median with interquartile range. PE, pulmonary thromboembolism; BMI, Body mass index; DVT, deep vein thrombosis

Table 2 shows the initial clinical manifestations of PE in both groups. PE-related cardiac distress was less frequent in patients with IPE compared to those with symptomatic PE (RV dysfunction: 0% vs. 18.7%, p<0.001; NT-proBNP: 1053.9 ± 2793.6 pg/mL vs. 2478.4 ± 6319.0 pg/mL, p = 0.043, respectively). Cardiac enzyme levels did not differ between the two groups. On chest CT, thromboembolism involvement of bilateral pulmonary arteries was significantly less prevalent in the IPE group than the symptomatic PE group (37.6% vs. 53.7%, p<0.001). There was no significant difference in the incidence of lung infarction between the two groups (4.6% vs. 6.6%, p = 0.378). IPE was more frequently located in the distal pulmonary artery, but this was not statistically significant. Coexisting DVT was more common in the symptomatic PE group than those in the IPE group (79.3% vs. 55.3%, p<0.001).

**Table 2:** Clinical manifestation of PE.

	Suspected PE (*n* = 229)	Incidental PE (*n* = 474)	Total (*n* = 703)	*P*-value
**Laboratory finding**^**¶**^				
D-dimer (ug/mL)	16.4 ± 16.5	12.3 ± 12.0	15.3 ± 15.5	0.027
Hemoglobin (g/dL)	11.1 ± 1.9	11.5 ± 1.8	11.3 ± 1.8	0.008
Platelet (x103/uL)	194.3 ± 110.1	226.6 ± 109.9	215.4 ± 111.0	<0.001
NT-proBNP (pg/mL)	2478.4 ± 6319.0	1053.9 ± 2793.6	2168.0 ± 5761.5	0.043
CK-MB (ng/mL)	5.0 ± 14.7	2.7 ± 6.1	4.5 ± 13.3	0.128
Troponin I (ng/mL)	0.7 ± 3.2	1.2 ± 7.3	0.8 ± 4.4	0.629
**Echocardiographic finding**^**¥**^				
RV dysfunction, n (%)	17 (18.7%)	0 (0.0%)	17 (10.3%)	<0.001
Dilated RV, n (%)	12 (13.2%)	3 (4.1%)	15 (9.1%)	0.079
D-shaped LV, n(%)	12 (13.2%)	3 (4.1%)	15 (9.1%)	0.079
RVSP, (%)	37.5 ± 13.5	30.6 ± 7.4	34.4 ± 11.7	<0.001
**CT finding**				
Distal*, n (%)	87 (38.0%)	201 (42.4%)	288 (41.0%)	0.301
Bilateral involvement, n (%)	123 (53.7%)	178 (37.6%)	301 (42.8%)	<0.001
Lung infarction, n (%)	15 (6.6%)	22 (4.6%)	37 (5.3%)	0.378
**Coexisting DVT**^**†**^	92 (79.3%)	47 (55.3%)	139 (69.2%)	<0.001
Distal DVT	34 (29.3%)	18 (21.2%)	52 (25.9%)	
Proximal DVT	58 (50.0%)	29 (34.1%)	87 (43.3%)	0.001

^**¶**^Laboratory test was conducted in all enrolled patients except NT-proBNP (140 patients of suspected PE and 39 patients of IPE) and cardiac enzyme (151 patients of suspected PE and 44 patients of IPE).

^**¥**^Echocardiography was conducted in 91 patients of suspected PE and 74 patients of IPE.

*Distal PE includes filling defects in segmental or subsegmental pulmonary artery.

^**†**^DVT is confirmed using Doppler ultrasonography or computed tomography. Proximal DVT was defined as occurring in the popliteal vein and above.

Data are presented as n (%) or median with interquartile range. PE, pulmonary thromboembolism; NT-proBNP, N-terminal pro blood natriuretic peptide; CK-MB, creatine kinase myocardial band; RV, right ventricle; RVSP, right ventricular systolic pressure; LV, left ventricle; DVT, deep vein thrombosis

Treatment with anticoagulation was less frequent in the IPE group than symptomatic PE group (52.3% vs. 83.0%, p<0.001). Details on PE treatment are provided in Table 3. Thrombolysis and surgical thrombectomy were rarely performed in patients with cancer-related PE. In multivariate Cox-regression analysis, metastatic cancer and cancer progression were independently associated with poor prognosis in IPE patients (adjusted HR with 95% confidence interval; 2.36 [1.67–3.34], p<0.001 and 2.08 [1.59–2.72], p<0.001, respectively). Administration of anticoagulants was significantly associated with reduced mortality in cancer patients (0.75 [0.57–0.97], p = 0.031). The effect of anticoagulation on the overall survival was consistent in patients with the proximal IPE (0.66 [0.47–0.92], p = 0.015), but not in patients with the distal IPE (1.07 [0.70–1.65], p = 0.740).

**Table 3:** Treatment of PE.

	Suspected PE (*n* = 229)	Incidental PE (*n* = 474)	Total (*n* = 703)	*P*-value
PE treatment	190 (83.0%)	248 (52.3%)	438 (62.3%)	< 0.001
Anticoagulation				< 0.001
VKA	127 (66.8%)	141 (56.9%)	268 (61.2%)	
LMWH	56 (29.5%)	100 (40.3%)	156 (35.6%)	
NOAC	5 (2.6%)	7 (2.8%)	12 (2.7%)	
Thrombolysis*	1 (0.4%)	-	1 (0.4%)	
Thrombectomy*	1 (0.4%)	-	1 (0.4%)	

*All patients who treated with thrombolysis or thrombectomy received anticoagulation therapy

Data are presented as n (%) or median with interquartile range. PE, pulmonary thromboembolism; VKA, vitamin K antagonist; LMWH, low molecular weight heparin; NOAC, new oral anticoagulant

**Fig 2:**
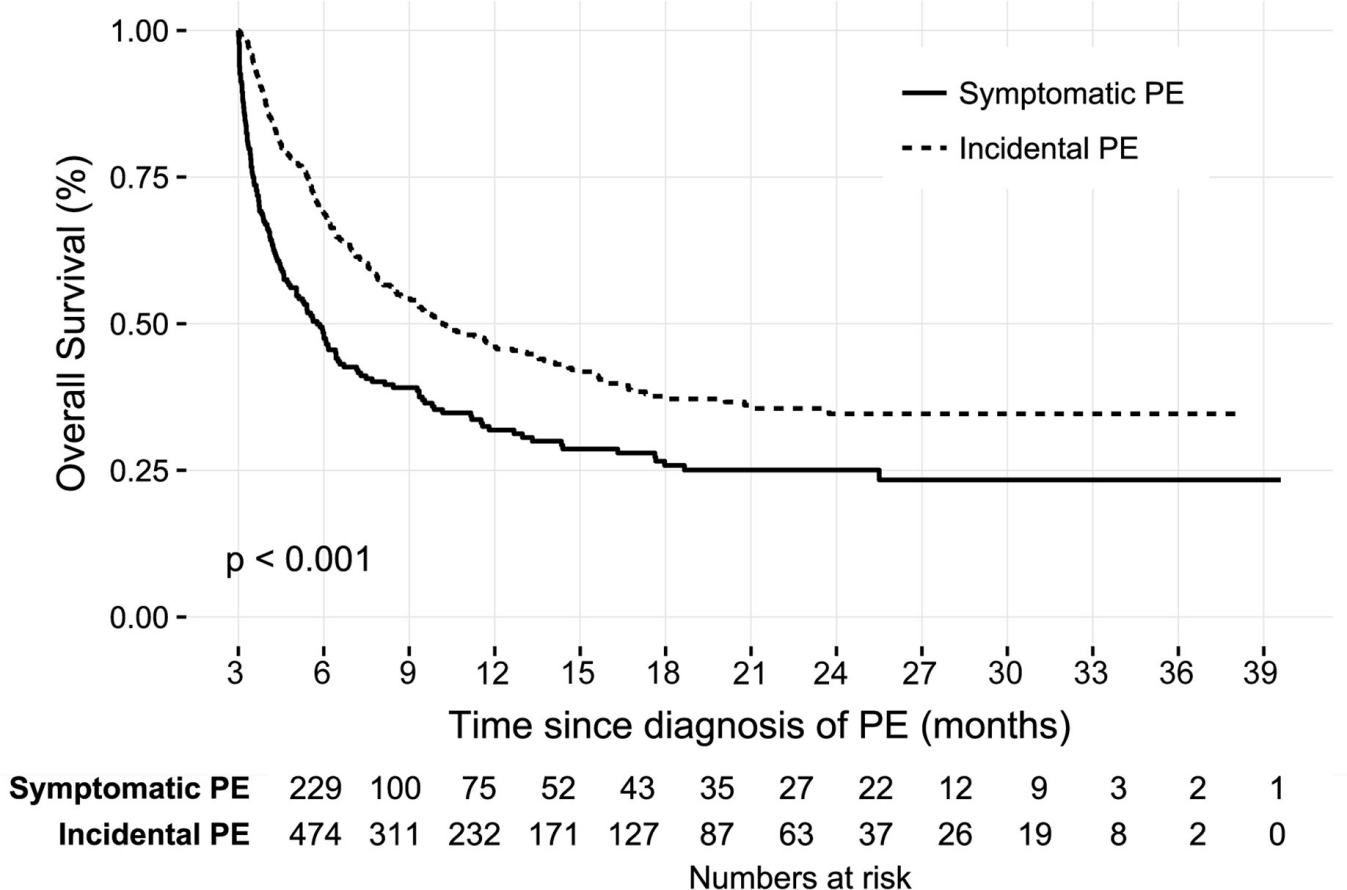
Kaplan-Meier survival curve comparing symptomatic and incidental PE in cancer patients. Abbreviations: PE, pulmonary embolism.

**Fig 3:**
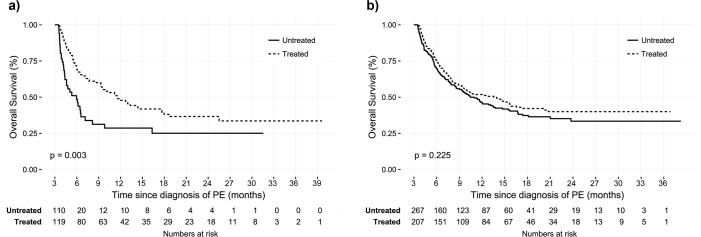
Clinical outcome for PE with or without treatment in cancer patients. (a) Kaplan-Meier survival curve for symptomatic PE with or without treatment. (b) Kaplan-Meier survival curve for incidental PE with or without treatment. Abbreviations, same as Fig 2.

**Fig 4:**
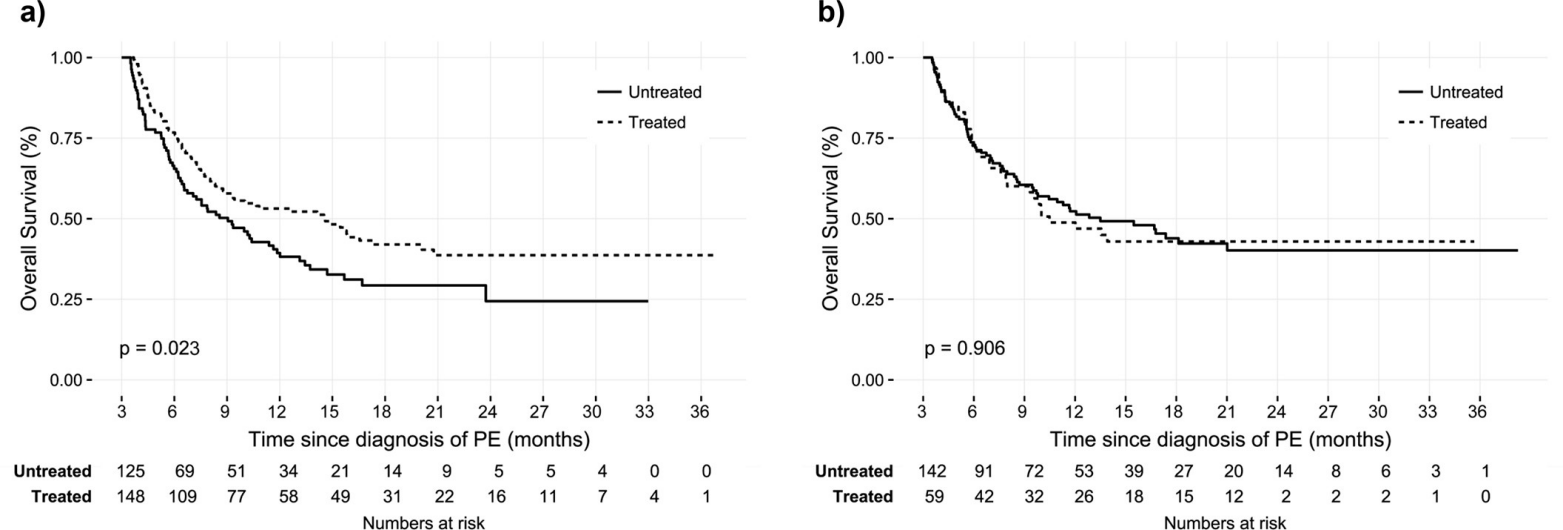
Clinical outcome for incidental PE with or without treatment in cancer patients. (a) Kaplan-Meier survival curve for proximally located incidental PE with or without treatment. (b) Kaplan-Meier survival curve for distally located incidental PE with or without treatment. Abbreviations, same as Fig 2.

Patients with IPE showed better overall survival than those with symptomatic PE (median survival 14.5 vs. 5.6 months, p<0.001) (Fig 2). There was significant difference in overall survival between the symptomatic PE patients with and without anticoagulation (Fig 3A). However, there was no prognostic difference in IPE patients according to the anticoagulation (Fig 3B). Subgroup analysis was performed to determine whether this lack of treatment benefit was consistent among different clot burdens. Patients with IPE who had proximal thromboembolism demonstrated significant improvement of overall survival with anticoagulation (median survival 12.2 vs. 23.4 months, p = 0.023) (Fig 4A). However, IPE patients with distally located thromboembolism did not show improved survival with anticoagulation (Fig 4B). Additionally, there was no difference of overall survival in IPE patients who had segmental or subsegmental thromboembolism (S1 Fig). Clinically relevant bleeding events occurred in 13.5% of patients who treated with anticoagulant (9.6% major bleeding and 3.9% minor bleeding, respectively).

Symptomatic VTE recurrence occurred in 3.6% of enrolled patients. There was no statistically significant difference in the rate of VTE recurrence according to presence of PE related symptoms (3.1% vs 3.8%, p = 0.780), treatment with anticoagulation (3.4% vs 3.8%, p = 0.974) or proximal PE (4.1% vs 2.8%, p = 0.471) between two groups. In patients with distal IPE, anticoagulation did not reduce the recurrent VTE compared to those without anticoagulation (3.7% vs 2.0%, p = 0.618).

## Discussion

In this retrospective cohort study, the clinical characteristics of IPE and symptomatic PE, as well as the prognostic impact according to anticoagulation, were investigated in patients with active cancer. Patients with cancer-related IPE tended to have more benign clinical presentation than those with symptomatic PE. While cancer patients with proximally located IPE showed survival benefit with anticoagulation, there was no significant difference of overall survival according to the anticoagulation in those with distally located IPE. Although several small-scaled retrospective studies suggested that there is no need for anticoagulation in distal IPE, prospective trials to confirm these issues have not been conducted with ethical issues. In consistent with our data, some studies demonstrated that distal IPE was not associated with poor prognosis and had better prognosis with no anticoagulation in cancer patients [3,10,11]. These previous studies suggested that because distal PE is likely to arise from a small DVT, the risk of progressive or recurrent VTE without anticoagulation is expected to be lower than in patients with a larger PE. However, there were too few enrolled patients in the previous studies to draw clear consensus. Moreover, most of the previous studies were focused on subsegmental isolated IPE only [3, 10, 11]. Our data demonstrated that anticoagulation did not affect mortality in cancer-related distal IPE, which was consistent across the subgroups with segmental or subsegmental PE. Taken together, the present study, with large number of patients, is meaningful in that it provides the evidence for the needs of prospective trials to access appropriate anticoagulation therapy in cancer patients with IPE.

Recent studies have reported that about 5% of patients with active cancer have IPE; among these, more than half showed filling defects in the distal pulmonary arteries [3, 11, 12]. Although IPE is commonly encountered at initial presentation for cancer or during follow up, its clinical presentation, natural course, and prognosis remain poorly understood. While symptomatic PE adversely impacts on survival among cancer patients [12], information regarding IPE outcome is limited. In contrast to our data, some researchers have reported that distal IPE in cancer patients is not a benign condition, and rates of recurrent VTE events are comparable to those in cancer patients with symptomatic PE [13]. However, these studies did not show the survival benefit of anticoagulation in distal IPE. The number and homogeneity of enrolled patients in our trial allows more reliable and definite conclusions than the previous studies.

Our data showed that a large number of patients presented with IPE rather than the suspected PE (about 2:1), which was a higher frequency than previously reported. This was likely to be related to the increased detection rate of pulmonary emboli which attributed by high resolution CT and largely inclusion of lung cancer patients.

Although the treatment rate of IPE in cancer patients was significantly lower than in those with symptomatic PE, the treatment rate of symptomatic PE itself was also relatively low (83%). There are several possible explanations for these findings. First, the high bleeding risk associated with bone marrow suppression as well as the poor functional status of cancer patients hampers the use of anticoagulants in patients with cancer. Second, concern regarding potential interaction between anticoagulants and anticancer drugs or relatively short expected survival may cause physicians to waver in treating PE in patients with cancer. Therefore, in real world practice, the actual anticoagulation rate of IPE in cancer patients was much lower than in those with symptomatic PE, especially for distally located IPE [8].

The reason why such clinical practices are not wrong is as follows. Firstly, although current guidelines suggest that IPE should receive similar initial and long-term anticoagulation therapy as symptomatic PE, regardless of location and extent of thrombus [7, 14], direct evidence regarding the treatment of distally located IPE is scarce. Therefore, they described as the evidence supporting their recommendations for isolated distal IPE is low quality and management should be tailored according to risk and benefit of each patient. Secondly, the advent of CT techniques left physicians with a large volume of small burden IPEs which have completely different nature from the PE detected in early studies. Our data included relatively large number of untreated patients with distal IPE compared to the previously reported data; nevertheless, mortality from PE or VTE recurrence was very rare in this group. Given the debate regarding the clinical relevance of distal IPE, the clinical significance and management of distal IPE should be reconsidered. We believe that our findings provide evidence to plan future randomized studies on the effectiveness and safety of anticoagulation versus no intervention in cancer patients with IPE under the careful monitoring.

### Study limitation

The current study has some limitations. First, this was a single-center observational study; thus, our results might be subject to unrecognized confounding factors. Although we performed multivariate analysis and adjusted for several potential confounding factors, we could not correct for unmeasured or unobserved variables. Second, this study was retrospective. However, a randomized controlled trial comparing PE with and without treatment has been deemed unethical because of the belief that PE left untreated is fatal. The only randomized trial that left PE patients without anticoagulation aborted prematurely because of markedly increased mortality without treatment. Despite retrospectively collected data, a large number of patients was the strength of our study. Lastly, our primary outcome only includes all-cause mortality rather than PE specific outcomes such as mortality due to PE and symptomatic VTE recurrence. Addition of these PE related outcomes to survival analysis would have provided more clinically meaningful insights about cancer related PE. However, small number of events prohibited us from drawing any conclusion on PE specific outcome with or without treatment from this retrospective study.

## Conclusions

In cancer patients who were diagnosed with IPE, the overall survival was different according to the embolic burden and anticoagulation therapy. Our data support the need for future prospective studies to identify which groups of cancer patients require anticoagulation treatment.

## Supporting information

S1 Fig Kaplan-Meier survival curves for distally located incidental PE with or without treatment in cancer patients. (a) Kaplan-Meier survival curve for segmental PE with or without treatment. (b) Kaplan-Meier survival curve for subsegmental PE with or without treatment. Abbreviations, same as Fig 2.
(TIF)

S1 File Pulmonary embolism in cancer patients dataset. (XLSX)
