# Supplementary figures and images for: Does anticoagulation needed for distally located incidental pulmonary thromboembolism in patients with active cancer?
Source: PLoS One. 2019 Sep 12;14(9):e0222149. doi: 10.1371/journal.pone.0222149 (PMC6742487; doi:10.1371/journal.pone.0222149)

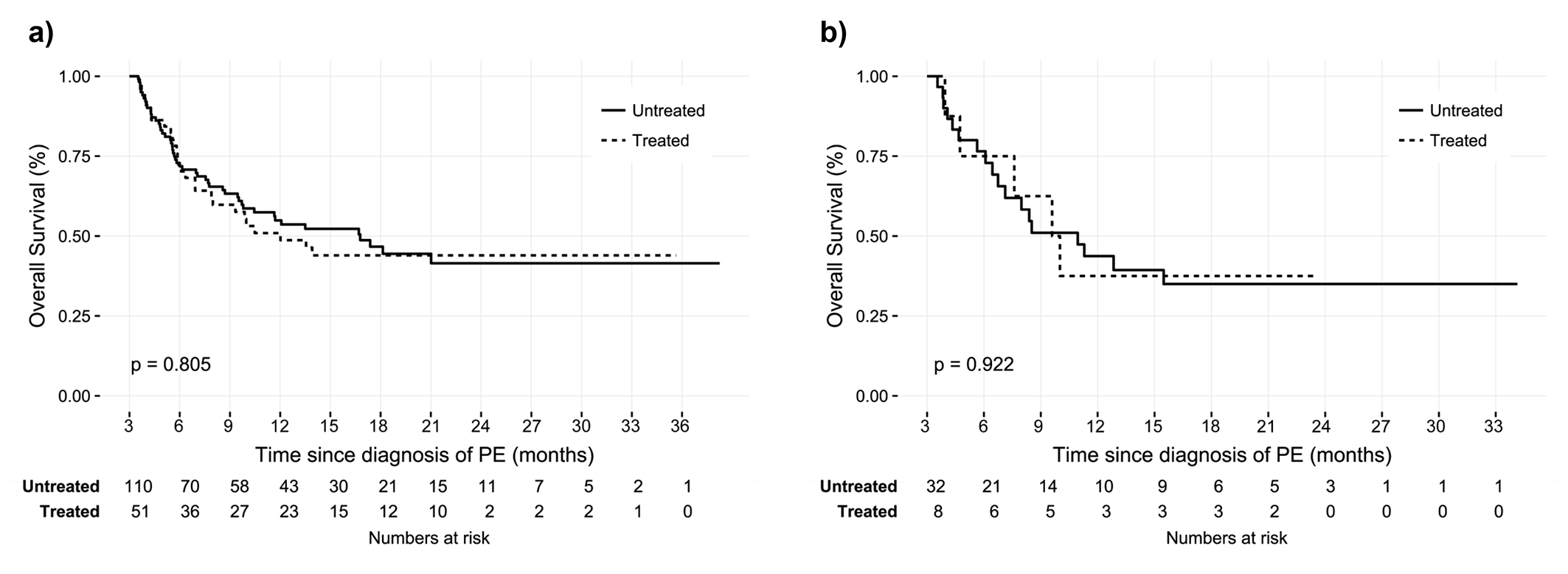

Supplement: S1 Fig — (a) Kaplan-Meier survival curve for segmental PE with or without treatment. (b) Kaplan-Meier survival curve for subsegmental PE with or without treatment. Abbreviations, same as Fig 2. (TIF) [file pone.0222149.s001.tif]
